# A systems view of type 2 diabetes-associated metabolic perturbations in saliva, blood and urine at different timescales of glycaemic control

**DOI:** 10.1007/s00125-015-3636-2

**Published:** 2015-06-07

**Authors:** Noha A. Yousri, Dennis O. Mook-Kanamori, Mohammed M. El-Din Selim, Ahmed H. Takiddin, Hala Al-Homsi, Khoulood A. S. Al-Mahmoud, Edward D. Karoly, Jan Krumsiek, Kieu Thinh Do, Ulrich Neumaier, Marjonneke J. Mook-Kanamori, Jillian Rowe, Omar M. Chidiac, Cindy McKeon, Wadha A. Al Muftah, Sara Abdul Kader, Gabi Kastenmüller, Karsten Suhre

**Affiliations:** Department of Physiology and Biophysics, Weill Cornell Medical College in Qatar, Qatar Foundation – Education City, PO Box 24144, Doha, Qatar; Department of Computer and Systems Engineering, Alexandria University, Alexandria, Egypt; Department of Clinical Epidemiology, Leiden University Medical Centre, Leiden, the Netherlands; Department of Endocrinology, Leiden University Medical Centre, Leiden, the Netherlands; Dermatology Department, Hamad Medical Corporation, Doha, Qatar; Metabolon Inc., Durham, NC USA; Institute of Computational Biology, Helmholtz Zentrum München, German Research Center for Environmental Health, Neuherberg, Germany; Clinical Research Core, Weill Cornell Medical College in Qatar, Qatar Foundation – Education City, Doha, Qatar; Institute of Bioinformatics and Systems Biology, Helmholtz Zentrum München, German Research Center for Environment Health, Neuherberg, Germany; German Center for Diabetes Research (DZD), Neuherberg, Germany

**Keywords:** Arab population, Asian population, Blood metabolomics, Gaussian graphical modelling, Glycaemic control, Metabolic dysregulation, Partial correlation, Saliva metabolomics, Systems biology, Type 2 diabetes, Urine metabolomics

## Abstract

**Aims/hypothesis:**

Metabolomics has opened new avenues for studying metabolic alterations in type 2 diabetes. While many urine and blood metabolites have been associated individually with diabetes, a complete systems view analysis of metabolic dysregulations across multiple biofluids and over varying timescales of glycaemic control is still lacking.

**Methods:**

Here we report a broad metabolomics study in a clinical setting, covering 2,178 metabolite measures in saliva, blood plasma and urine from 188 individuals with diabetes and 181 controls of Arab and Asian descent. Using multivariate linear regression we identified metabolites associated with diabetes and markers of acute, short-term and long-term glycaemic control.

**Results:**

Ninety-four metabolite associations with diabetes were identified at a Bonferroni level of significance (*p <* 2.3 × 10^−5^), 16 of which have never been reported. Sixty-five of these diabetes-associated metabolites were associated with at least one marker of glycaemic control in the diabetes group. Using Gaussian graphical modelling, we constructed a metabolic network that links diabetes-associated metabolites from three biofluids across three different timescales of glycaemic control.

**Conclusions/interpretation:**

Our study reveals a complex network of biochemical dysregulation involving metabolites from different pathways of diabetes pathology, and provides a reference framework for future diabetes studies with metabolic endpoints.

**Electronic supplementary material:**

The online version of this article (doi:10.1007/s00125-015-3636-2) contains peer-reviewed but unedited supplementary material, which is available to authorised users.

## Introduction

Metabolomics [1, 2] has been successfully used to identify molecules associated with diabetes [3], including metabolites from the three major energy sources (carbohydrates, lipids and proteins [4–6]) as well as molecules associated with plasma phospholipids [7, 8] and branched chain amino acids (BCAAs) [9, 10], and individual molecules such as α-hydroxybutyrate [11] and 2-aminoadipic acid [12]. To date, most large-scale, population-based studies have focused only on metabolites collected from a single biofluid, primarily blood or urine. However, we recently showed that saliva can also be used to identify metabolic changes in diabetes [13]. Since metabolic readouts of diabetes-related biochemical processes in circulating body fluids are primarily proxies for biochemical processes occurring elsewhere in the body, these results need to be interpreted in context. We propose that a systems-wide analysis combining metabolomic measurements obtained across different biofluids isolated from the same patient would improve our understanding of the interactions between and roles of different organs and tissues in the development and progression of diabetes.

Impaired glucose metabolism is a hallmark of diabetes, and episodes of dysregulated glucose levels can be monitored on different timescales. For studies assessing the associations between metabolites and diabetes in a case–control design, it is essential to interrogate metabolites that are specifically associated with individual markers of glycaemic control in patients with diabetes. The most frequently used endpoints for medically assessing patients with diabetes is the blood HbA_1c_ level, which reflects the time-averaged blood glucose level collected over the previous 2–3 months [14] and can be considered a marker of long-term glycaemic control. The 1,5-anhydroglucitol (1,5-AG) level is also used as a marker of time-averaged blood glucose levels, with lower levels of 1,5-AG being the consequence of frequent episodes of glucosuria experienced over the previous 1–2 weeks [13, 14]. Finally, glucose in the urine (glucosuria) is used as a marker of acute glucose dysregulation over a timescale of 6–12 h. Therefore, for the purpose of this study, glucose homeostasis in individuals shall be characterised on three different timescales, with urinary glucose serving as a marker of acute glycaemic control, plasma 1,5-AG levels as a marker of short-term glycaemic control and HbA_1c_ as a marker of long-term glycaemic control.

Gaussian graphical models (GGMs) have proven to be powerful tools for detecting signatures of biochemical pathways in large metabolomics datasets [15, 16]. Using this approach, the variability in metabolic individuality encountered in larger population studies represents a natural experiment that allows one to derive biochemical connections between correlated metabolites in a purely data-driven manner. Metabolite–metabolite interactions in these GGMs are identified by partial correlations between the measured metabolites; they have been shown to correspond to known biochemical interactions that can be used for reconstructing metabolic networks from data alone [16, 17]. Mapping metabolite–disease associations onto such networks may then allow for functional interpretation in a naturally derived biochemical context [18–20].

In this study, we examined how metabolic systems are altered in diabetes and how these changes are related to glycaemic control over three different timescales (acute, short term and long term) across three biofluids (plasma, urine and saliva). Using a comprehensive non-targeted metabolomics approach, we made over 2,000 individual metabolite measures per individual in plasma, urine and saliva samples from 369 participants of Arabic and Asian ethnicities. Using linear regression analysis with relevant covariates and stringent Bonferroni correction, we first identified metabolites in saliva, plasma and urine that were associated with diabetes. Among these metabolites, we then identified those associated with at least one of the three glycaemic control variables in samples from patients with diabetes. Finally, we derived a GGM for all metabolites measured in all three biofluids, thereby creating a biochemical reference network that revealed biochemical connections between all diabetes-associated metabolites across the different biofluids and timescales of glycaemic control.

## Methods

### Study design

This study was embedded in the Qatar Metabolomics Study on Diabetes (QMDiab), a cross-sectional case–control study with 374 participants [13, 20]. All study participants were enrolled between February 2012 and June 2012 at the Dermatology Department of Hamad Medical Corporation (HMC) in Doha, Qatar. Inclusion criteria were a primary form of type 2 diabetes (for patients) or an absence of type 2 diabetes (for controls). Sample collection was conducted in the afternoon, after the general operating hours of the morning clinic. Patient and control samples were collected in a random order as they became available and at the same location using identical protocols, instruments and study personnel. Samples from patients and controls were processed in the laboratory in a blinded manner. Data from five participants were excluded from the analysis because of incomplete records, leaving 176 patients and 193 controls. Of the 193 control participants initially enrolled, 12 had HbA_1c_ levels above 6.5% (48 mmol/mol) and were subsequently classified as patients, resulting in 188 patients and 181 controls.

### Ethics statement

This study was conducted following the World Medical Association Declaration of Helsinki – Ethical Principles for Medical Research Involving Human Subjects. It was approved by the Institutional Review Boards of HMC and Weill Cornell Medical College – Qatar (WCMC-Q; research protocol no. 11131/11). All study participants provided written informed consent.

### Phenotyping

Information regarding age, sex, ethnicity, BMI and diabetes history was obtained by trained researchers using questionnaires and standardised protocols (Table 1). Saliva, plasma and urine specimens were collected and processed using standardised collection protocols and stored on ice for transportation. Within 6 h of collection, all samples were clarified by centrifugation at 2,500 *g* for 10 min, aliquoted and stored at −80°C. Duplicate blood samples were sent directly to the hospital’s clinical biochemistry laboratory for comprehensive analysis including HbA_1c_ level, lipid profile, general chemistry and a complete blood count [13].

**Table 1:** General characteristics of the participants

Characteristic	Type 2 diabetes (*n* = 188)	Controls (*n* = 181)	*p* value
Age (years)	53.8 (35.0–70.7)	38.5 (23.6–62.3)	<0.001
Sex (female [%])	81 (43.1)	99 (54.7)	0.03
Ethnicity^a^ (%)			
Arab	93 (43.1)	113 (62.4)	
South Asian	74 (39.4)	39 (21.5)	0.002
Filipino	14 (7.4)	22 (12.2)	
Other or mixed	7 (3.7)	7 (3.9)	
BMI (kg/m^2^)	29.5 (21.6–42.6)	27.6 (21.7–39.1)	0.004
Waist circumference (cm)	101.0 (83.0–128.0)	94.5 (74.0–116.1)	<0.001
Hypertension (%)	103 (54.8%)	27 (14.9%)	<0.001
Total cholesterol (mmol/l)	4.95 (3.03–6.88)	5.13 (3.74–6.61)	0.11
HDL-cholesterol (mmol/l)	1.13 (0.71–1.78)	1.22 (0.77–1.90)	0.02
LDL-cholesterol (mmol/l)	2.79 (1.45–4.45)	3.07 (1.55–4.67)	0.02
Triacylglycerol (mmol/l)	1.77 (0.76–4.69)	1.38 (0.63–3.61)	0.002
Creatinine (μmol/l)	75.0 (48.4–112.6)	69.0 (50.0–99.0)	0.01
HbA_1c_ (%)	7.8 (5.6–11.5)	5.5 (4.7–6.2)	<0.001
HbA_1c_ (mmol/mol)	62 (38–102)	37 (28–44)	<0.001
Duration of diabetes (years)	8.0 (1.0–31.7)	N/A	N/A
Diabetes medication (%)			
Insulin	39 (20.7)	0 (0.0)	N/A
Oral hypoglycaemic medication			
Metformin	120 (63.8)	0 (0.0)	N/A
Sulfonylureas	70 (37.2)	0 (0.0)	N/A
Thiazolidinediones	6 (3.2)	0 (0.0)	N/A
Dipeptidyl peptidase-4 inhibitors	19 (10.1)	0 (0.0)	N/A
Other	15 (8)	0 (0.0)	N/A
Oral corticosteroids	6 (3.2)	1 (0.6)	0.12

Data represent median (90% range) or number of participants (%)

*p* values are based on the Mann–Whitney *U* or *χ* ^2^ test

^a^Classified as Arabs (from Bahrain, Egypt, Iraq, Jordan, Kuwait, Lebanon, Morocco, Oman, Palestine, Qatar, Saudi Arabia, Somalia, Sudan, Syria, Tunisia, United Arab Emirates and Yemen) or as South Asians (from Bangladesh, India, Nepal, Pakistan and Sri Lanka)

N/A, not applicable

### Metabolomics

Metabolic profiling was achieved using ultra-HPLC and GC separation, coupled with tandem MS using established procedures and technology (at Metabolon, Durham, NC, USA; Table 2) [21, 22]. The essential steps of this process are provided as electronic supplementary material (ESM) Methods. Median process variability, as determined by repeated measurements of pooled samples, was 15.3% in saliva, 15.8% in plasma and 9.8% in urine. In the initial sample set of 374 participants, 147 metabolites were detected in saliva, plasma and urine, 391 were detected in only two sample types and 1,030 were detected in a single sample type. Thus, a total of 2,253 individual metabolite signals were measured in the three biofluids (603 in saliva, 759 in plasma and 891 in urine) when counting the same molecule in different biofluids as separate entities, or a total of 1,568 unique metabolites when counting detection of the same molecule in multiple fluids only once. After excluding metabolite measures with fewer than 50 valid detections in a single fluid (13.6%), many of which were xenobiotics related to medication, 2,178 distinct metabolite measures were used for analysis (ESM Table 1).

**Table 2:** Number of samples and metabolites detected

Sample^a^	Participants (*n*)	Metabolites (*n*)
Saliva	328	581
Plasma	359	720
Urine	356	877
Total	1,043	2,178

^a^At least one type of sample was collected from each of the 369 study participants. Reasons for missing samples are that some patients did not provide blood or urine; in some cases, no saliva could be collected because of technical problems with the collection kit

### Statistical analysis

#### Regression analysis

Metabolite levels were scaled by run-day medians, normalised using osmolality (saliva and urine data only), log-transformed and then *z*-scored. Missing values in metabolites with more than 20% missing data points were imputed to the smallest detected value since it can be assumed that they are probably below the detection limit of the method. Values for metabolites >4 SD from the mean were excluded from the analysis. Multivariate linear regression, adjusting for age, sex, ethnicity and BMI, was used to assess the statistical significance of the association of metabolites with diabetes, as previously described [5]. A stringent Bonferroni level of significance of *p <* 2.3 × 10^−5^ (=0.05/2,178) was used to infer association.

#### Glycaemic control

By limiting the analysis to Bonferroni significant diabetes-associated metabolites (*n* = 94), we examined their association with acute glycaemic dysregulation (6–12 h) and short- (1–2 weeks) [13] and long-term (2–3 months) [14] glycaemic control; only diabetes patients were included in this case. Acute glycaemic dysregulation was defined by MS detection of glucose in urine (66 out of 188 cases; a dichotomous variable). Note that metabolomics measurements only provide semiquantitative measures of glucose in urine. Therefore, a physiological cut-off to define glucosuria could not be applied. However, in only two of the 181 controls was glucose detected in urine. We therefore consider the detection limit of the MS measure a viable proxy. Short- and long-term glycaemic control scales were defined by 1,5-AG and HbA_1c_ levels in plasma, respectively (continuous variables) [14]. As in the previous regression analysis, multivariate linear regression adjusting for age, sex, ethnicity and BMI was performed. A Bonferroni level of significance of *p <* 1.8 × 10^−4^ (=0.05/(94 × 3)) was used to infer association (94 metabolites and three measures of glycaemic control).

#### GGMs

**Fig. 1:**
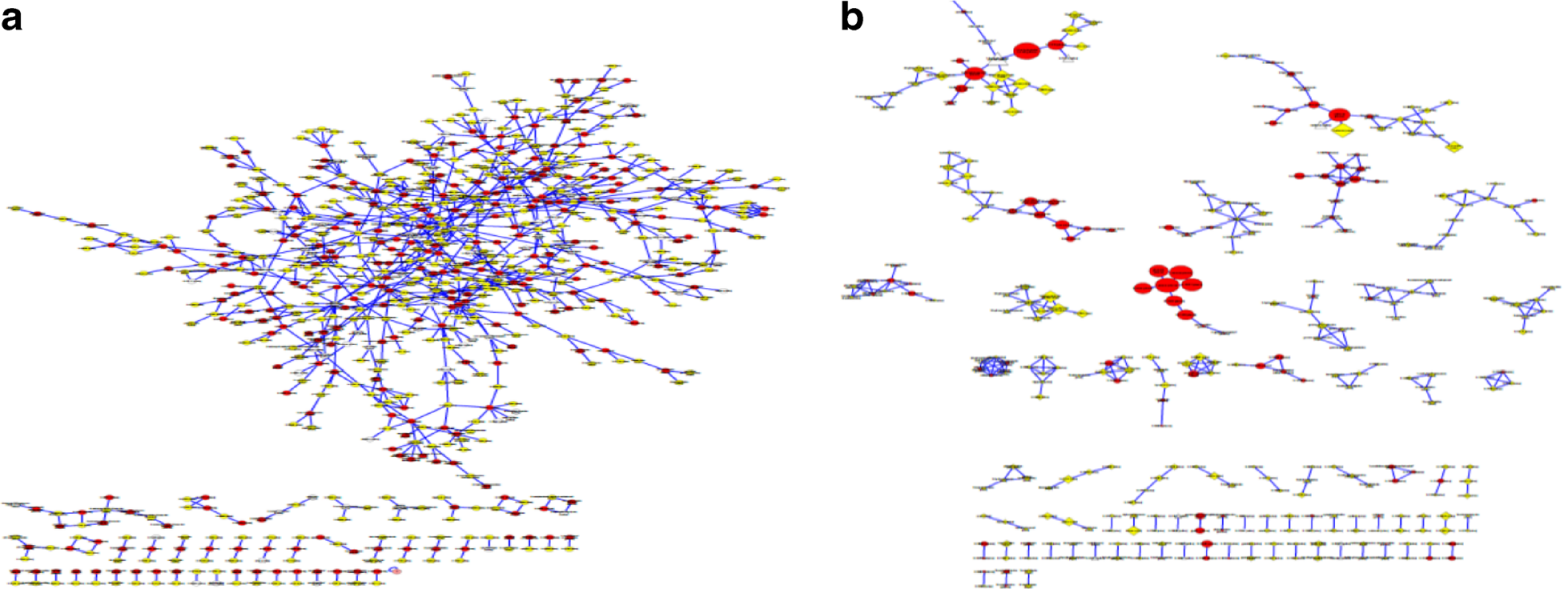
Workflow for the generation of the GGM. Starting with 2,178 metabolites and 2.3 million partial correlations, two steps were conducted. (**a**) Step 1: filtering on significant partial correlations (3,742) by removing metabolites with no significant correlation to any other metabolite, leaving 1,907 metabolites in the GGM network. (**b**) Step 2: filtering on metabolites nominally associated with type 2 diabetes (*p* < 0.05), i.e. 546 metabolites, resulted in 33 subnetworks containing at least three metabolites and covering 243 metabolites

Based on the complete quality-checked and imputed metabolomic datasets (369 individuals and 2,178 metabolite measures), we computed partial correlation values adjusting for diabetes state, age, sex, ethnicity and BMI to construct the GGMs. A stringent Bonferroni level of significance of *p <* 2.1 × 10^−8^ [=0.05/([2,178 × 2,177]/2)] was applied to determine significant partial correlation edges. In the resulting GGM with 3,742 edges (significant partial correlations) connecting each of 1,907 metabolites with at least one other metabolite, we only kept the 546 metabolites nominally associated (*p <* 0.05) with diabetes and removed all other metabolites with their edges. Thus, a total of 33 GGM subnetworks (with at least three metabolites in a network) were obtained (Fig. 1). All statistical analyses were performed using the R statistical package (version 2.14, www.r-project.org/) and the GeneNet package in R (http://cran.r-project.org/web/packages/GeneNet/).

## Results

### Of 2,178 metabolite measures in saliva, plasma and urine, 94 were associated with diabetes at a Bonferroni level of significance

Of the 2,178 individual metabolite associations in the three biofluids tested, 546 displayed nominal significance (*p <* 0.05) with diabetes after adjusting for covariates (Table 3 and ESM Table 2). Ninety-four of the 546 metabolite associations remained significant after stringent Bonferroni correction (*p <* 2.3 × 10^−5^). These 94 associations were found across the three biofluids as follows: three in saliva, 28 in plasma and 63 in urine, with a total of 24 associations representing metabolites of unknown biochemical identity (unknowns). Eleven of the 94 associations were statistically significant in more than one biofluid. The 94 metabolite associations covered 59 distinct metabolites of known identity and 23 of unknown identity. Sixteen of the known and 22 of the unknown metabolites have not previously been reported in association with diabetes.

**Table 3:** Metabolites associated with type 2 diabetes

Metabolite^a^	Superpathway^b^	Pathway	Saliva		Plasma		Urine		Reported association with a diabetes-related phenotype^d^
			β^c^	Sig	β	Sig	β	Sig	
Alanine	Amino acid	Alanine and aspartate metabolism	0.196		0.110		0.541	†	[24]
*N*-acetyl-β-alanine	Amino acid	Alanine and aspartate metabolism	n.d.		0.188		0.517	†	Not previously reported
Creatinine	Amino acid	Creatine metabolism	n.d.		−0.103		−0.558	†	[5]
2-Hydroxybutyrate	Amino acid	Cysteine, methionine, SAM, taurine metabolism	0.158		0.703	†	0.881	†	[4, 6, 11, 33, 41]
Cysteine	Amino acid	Cysteine, methionine, SAM, taurine metabolism	n.d.		0.186		0.654	†	[6, 25]
α-Ketobutyrate	Amino acid	Cysteine, methionine, SAM, taurine metabolism	−0.032		0.592	†	n.d.		[11, 25]
Homocysteine	Amino acid	Cysteine, methionine, SAM, taurine metabolism	n.d.		n.d.		−0.639	†	[41, 42]
Pyroglutamine	Amino acid	Glutamate metabolism	−0.305	*	−0.471	†	−0.331	*	Not previously reported
Glutamate	Amino acid	Glutamate metabolism	0.119		0.209		−0.478	†	[43]
5-Oxoproline	Amino acid	Glutathione metabolism	0.241		−0.235		−0.753	†	[41]
β-Hydroxypyruvate	Amino acid	Glycine, serine and threonine metabolism	n.d.		0.986	†	0.909	†	Not previously reported
1-Methylhistidine	Amino acid	Histidine metabolism	n.d.		−0.593	†	−0.598	†	[44, 45]
*Trans*-urocanate	Amino acid	Histidine metabolism	0.121		n.d.		−0.533	†	[46]
Pipecolate	Amino acid	Lysine metabolism	0.281		0.697	†	0.822	†	[28, 41]
3-methoxytyrosine	Amino acid	Phenylalanine & tyrosine metabolism	n.d.		−0.526	†	−0.279	*	Not previously reported
4-Hydroxyphenylpyruvate	Amino acid	Phenylalanine & tyrosine metabolism	−0.219		0.490	*	0.581	†	Not previously reported
Vanillylmandelate	Amino acid	Phenylalanine & tyrosine metabolism	n.d.		n.d.		−0.651	†	Not previously reported
Homovanillate	Amino acid	Phenylalanine & tyrosine metabolism	n.d.		n.d.		−0.523	†	Not previously reported
Phenylalanine	Amino acid	Phenylalanine & tyrosine metabolism	−0.006		0.109		0.573	†	[5, 25]
Kynurenate	Amino acid	Tryptophan metabolism	n.d.		−0.322	*	−0.531	†	[47]
3-Hydroxyproline	Amino acid	Urea cycle; arginine-, proline-, metabolism	n.d.		n.d.		0.647	†	[48]
Citrulline	Amino acid	Urea cycle; arginine-, proline-, metabolism	0.148		−0.588	†	n.d.		[4, 5]
Homocitrulline	Amino acid	Urea cycle; arginine-, proline-, metabolism	n.d.		−0.207		−0.541	†	[5]
Ornithine	Amino acid	Urea cycle; arginine-, proline-, metabolism	0.077		−0.378	*	0.525	†	[41, 49]
Proline	Amino acid	Urea cycle; arginine-, proline-, metabolism	−0.010		0.196		0.654	†	[4, 5]
3-Hydroxyisobutyrate	Amino acid	Valine, leucine and isoleucine metabolism	n.d.		0.541	†	0.529	†	[50, 51]
α-Hydroxyisovalerate	Amino acid	Valine, leucine and isoleucine metabolism	−0.022		0.066		0.683	†	[51]
Isobutyrylcarnitine	Amino acid	Valine, leucine and isoleucine metabolism	0.203		−0.067		−0.532	†	[52, 53]
Isoleucine	Amino acid	Valine, leucine and isoleucine metabolism	0.090		0.179		0.589	†	[4, 5]
Leucine	Amino acid	Valine, leucine and isoleucine metabolism	−0.071		0.126		0.613	†	[4–6]
Fructose	Carbohydrate	Fructose, mannose, galactose, starch, and sucrose metabolism	−0.172		0.878	†	0.177		[4, 6]
Mannose	Carbohydrate	Fructose, mannose, galactose, starch, and sucrose metabolism	−0.091		1.136	†	0.731	†	[4, 5, 41]
1,5-AG	Carbohydrate	Glycolysis, gluconeogenesis, pyruvate metabolism	−0.998	†	−1.287	†	0.161		[4, 5, 13, 41, 54]
1,3-Dihydroxyacetone	Carbohydrate	Glycolysis, gluconeogenesis, pyruvate metabolism	0.140		0.631	†	n.d.		Not previously reported
Glucose	Carbohydrate	Glycolysis, gluconeogenesis, pyruvate metabolism	0.159		1.158	†	0.913	†	Diagnostic for diabetes
Lactate	Carbohydrate	Glycolysis, gluconeogenesis, pyruvate metabolism	0.063		0.474	*	0.584	†	[4, 24, 41, 55]
Pyruvate	Carbohydrate	Glycolysis, gluconeogenesis, pyruvate metabolism	0.188		0.718	†	−0.190		[23, 41]
Arabitol	Carbohydrate	Nucleotide sugars, pentose metabolism	−0.274		0.095		−0.546	†	Not previously reported
Gluconate	Carbohydrate	Nucleotide sugars, pentose metabolism	−0.249		0.949	†	0.185		Not previously reported
Ribose	Carbohydrate	Nucleotide sugars, pentose metabolism	0.007		n.d.		−0.603	†	Not previously reported
Xylonate	Carbohydrate	Nucleotide sugars, pentose metabolism	−0.112		0.079		−0.507	†	Not previously reported
Threonate	Cofactors and vitamins	Ascorbate and aldarate metabolism	−0.118		0.094		−0.539	†	[41]
2-Methylcitrate	Energy	Krebs cycle	n.d.		n.d.		−0.624	†	[56]
Malate	Energy	Krebs cycle	−0.103		0.303	*	0.715	†	[4]
7-Ketodeoxycholate	Lipid	Bile acid metabolism	n.d.		n.d.		−0.549	†	Not previously reported
Adipate	Lipid	Fatty acid, dicarboxylate	n.d.		n.d.		−0.584	†	[57]
Ethanolamine	Lipid	Glycerolipid metabolism	−0.046		n.d.		−0.546	†	[6, 41]
*Myo*-inositol	Lipid	Inositol metabolism	−0.140		0.126		0.887	†	[5, 11, 41]
3-Hydroxybutyrate	Lipid	Ketone bodies	−0.065		0.271	*	0.917	†	[5, 6, 41, 58]
Acetoacetate	Lipid	Ketone bodies	−0.053		n.d.		0.584	†	[59]
Heptanoate (7:0)	Lipid	Medium-chain fatty acid	−0.018		−0.577	†	n.d.		[4, 5]
N1-methyladenosine	Nucleotide	Purine metabolism, adenine containing	n.d.		−0.074		−0.495	†	Not previously reported
Pro-hydroxy-pro	Peptide	Dipeptide	n.d.		−0.548	†	−0.601	†	[5]
Glycylglycine	Peptide	Dipeptide	−0.175		n.d.		−0.621	†	Not previously reported
γ-Glutamylglutamine	Peptide	γ-Glutamyl	n.d.		−0.503	†	n.d.		[60]
γ-Glutamylleucine	Peptide	γ-Glutamyl	−0.093		−0.026		0.551	†	[5]
Benzoate	Xenobiotics	Benzoate metabolism	−0.109		−0.539	†	−0.276	*	[6]
Glycolate (hydroxyacetate)	Xenobiotics	Chemical	−0.232		0.086		−0.478	†	Not previously reported
Metformin	Xenobiotics	Drug	0.601	†	1.042	†	1.116	†	Diabetes medication
X-11333	Unknown	Unknown	n.d.		n.d.		−0.603	†	Not previously reported
X-10593	Unknown	Unknown	n.d.		n.d.		−0.554	†	Not previously reported
X-11315	Unknown	Unknown	−0.555	†	−0.820	†	n.d.		[4]
X-11429	Unknown	Unknown	n.d.		−0.824	†	−0.186		Not previously reported
X-11540	Unknown	Unknown	n.d.		−0.580	†	n.d.		Not previously reported
X-12170	Unknown	Unknown	n.d.		n.d.		−0.539	†	Not previously reported
X-12253	Unknown	Unknown	n.d.		n.d.		−0.557	†	Not previously reported
X-12682	Unknown	Unknown	n.d.		n.d.		0.583	†	Not previously reported
X-13431	Unknown	Unknown	n.d.		−0.225		−0.629	†	Not previously reported
X-13840	Unknown	Unknown	n.d.		n.d.		−0.520	†	Not previously reported
X-14331	Unknown	Unknown	n.d.		n.d.		0.741	†	Not previously reported
X-14625	Unknown	Unknown	n.d.		n.d.		0.750	†	Not previously reported
X-14955	Unknown	Unknown	n.d.		n.d.		0.665	†	Not previously reported
X-15497	Unknown	Unknown	−0.103		0.575	†	n.d.		Not previously reported
X-15503	Unknown	Unknown	n.d.		−0.261	*	−0.810	†	Not previously reported
X-17299	Unknown	Unknown	n.d.		−0.412	*	−0.560	†	Not previously reported
X-17323	Unknown	Unknown	n.d.		n.d.		−0.483	†	Not previously reported
X-17629	Unknown	Unknown	n.d.		−0.669	†	n.d.		Not previously reported
X-17676	Unknown	Unknown	n.d.		n.d.		−0.884	†	Not previously reported
X-18221	Unknown	Unknown	n.d.		1.011	†	n.d.		Not previously reported
X-18475	Unknown	Unknown	n.d.		n.d.		−0.627	†	Not previously reported
X-18887	Unknown	Unknown	−0.023		n.d.		−0.653	†	Not previously reported
X-19437	Unknown	Unknown	−0.060		−0.846	†	0.367	*	Not previously reported

^a^Limited to associations at a Bonferroni level of significance of *p <* 2.3 × 10^−5^ (†); nominal significant associations (*p <* 0.05) in other body fluids are included (*); metabolites not detected (n.d.) in the matrix are reported

^b^Metabolites are sorted by pathway classification

^c^Estimators of effect size (β) are expressed as differences in SD between patients and controls, using *z*-scored and log-scaled data; positive β values indicate higher metabolite concentrations in diabetes patients compared with controls

^d^Where available, previously published associations of these metabolites with a diabetes-related phenotype are cited

SAM, *S*-Adenosylmethionine; Sig, statistical significance

### Of the 94 diabetes associations, 65 were also identified as specifically associated with acute, short-term or long-term glycaemic control within the diabetes group

**Fig. 2:**
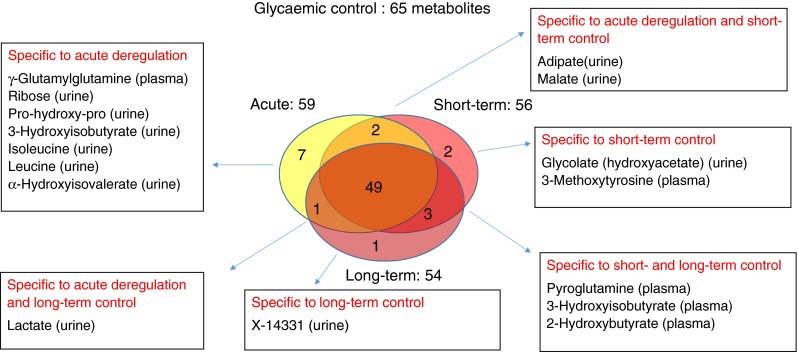
Venn diagram of metabolites specific to and overlapping with the three glycaemic control timescales

By limiting the analysis to the 94 diabetes–metabolite associations, and further to samples collected only from patients with diabetes, we identified 65 associations—at a Bonferroni level of significance (*p <* 1.8 × 10^−4^ = 0.05/[94 × 3])—with at least one of the three glycaemic control timescales investigated here: presence/absence of glucose in urine (glucosuria) as an acute marker; 1,5-AG in plasma as a short-term marker; and HbA_1c_ as a long-term marker of glycaemic control (Table 4). Among the 65 metabolite associations (one in saliva, 21 in plasma, 43 in urine), 59 were associated with glucosuria, 56 with 1,5-AG in blood plasma, 54 with HbA_1c_ and 49 with all three timescales (Fig. 2). Twenty-nine of the 94 diabetes–metabolite associations did not associate with any timescale of glycaemic control.

**Table 4:** Metabolites associated with the three timescales of glycaemic control

Timescale of dysregulation of glycaemic control^a^	Metabolite^b^	β			−log(*p*)			GGM subnetwork
		Acute	Short term	Long term	Acute	Short term	Long term	
Acute	3-Hydroxyisobutyrate (urine)	0.696	0.558	0.106	5.3^†^	2.3	2.0	Urinary ketone body
	Isoleucine (urine)	0.706	0.388	0.078	5.4^†^	1.3	1.2	Glycolysis–BCAAs
	Leucine (urine)	0.799	0.573	0.100	5.9^†^	2.0	1.6	Glycolysis–BCAAs
	α-Hydroxyisovalerate (urine)	0.810	0.637	0.116	6.6^†^	2.6	2.1	1,5-AG
	Pyruvate (plasma)	0.919	0.993	0.213	8.5^†^	6.3^†^	6.6^†^	Glycolysis–BCAAs
	Lactate (urine)	0.921	0.630	0.159	9.7^†^	2.9	4.2^†^	Urinary ketone body
	3-Hydroxybutyrate (urine)	1.056	1.182	0.248	12.5^†^	9.1^†^	9.7^†^	Urinary ketone body
	2-Hydroxybutyrate (urine)	1.165	1.138	0.211	15.4^†^	8.3^†^	6.8^†^	Urinary ketone body
	Acetoacetate (urine)	1.299	1.241	0.244	18.8^†^	9.6^†^	8.8^†^	1,5-AG
	Mannose (urine)	1.285	1.372	0.289	20.1^†^	12.7^†^	13.1^†^	1,5-AG
	β-Hydroxypyruvate (urine)	1.622	1.507	0.301	35.1^†^	14.1^†^	13.6^†^	1,5-AG
	Glucose (urine)	1.864	1.574	0.342	–	17.4^†^	19.8^†^	1,5-AG
Short term	Glycolate (hydroxyacetate) (urine)	−0.266	−0.828	−0.130	1.1	4.9^†^	2.9	Urinary ketone body
	3-Hydroxyisobutyrate (plasma)	0.437	0.908	0.155	2.3	5.7^†^	3.9^†^	Glycolysis–BCAAs
	α-Ketobutyrate (plasma)	0.607	1.050	0.185	4.2^†^	7.7^†^	5.5^†^	Glycolysis–BCAAs
	2-Hydroxybutyrate (plasma)	0.575	1.217	0.189	3.7	10.2^†^	5.6^†^	Glycolysis–BCAAs
	1,5-AG (saliva)	−1.159	−2.141	−0.327	7.3^†^	18.6^†^	6.4^†^	1,5-AG
	1,5-AG (plasma)	−1.222	−2.632	−0.432	16.3^†^	–	36.3^†^	1,5-AG
Long term	1,3-Dihydroxyacetone (plasma)	0.692	0.876	0.209	4.9^†^	5.0^†^	6.5^†^	1,5-AG
	Fructose (plasma)	1.048	1.296	0.317	11.6^†^	11.3^†^	15.9^†^	Carbohydrates
	β-Hydroxypyruvate (plasma)	1.263	1.561	0.351	16.2^†^	14.8^†^	18.0^†^	1,5-AG
	Gluconate (plasma)	1.081	1.290	0.338	13.2^†^	11.8^†^	18.9^†^	Carbohydrates
	Glucose (plasma)	1.362	1.686	0.389	21.3^†^	20.7^†^	26.4^†^	Carbohydrates
	Mannose (plasma)	1.282	1.743	0.389	18.9^†^	23.4^†^	27.5^†^	Carbohydrates

^a^Metabolites are grouped by the timescale that displayed the strongest association and sorted by *p* value

^b^A selection of 24 out of 65 significantly associated (Bonferroni level) metabolites is shown here, excluding unknown metabolites and limited to metabolites that are part of one of the four larger GGM subnetworks discussed in this paper. The full list is reported in ESM Table 3

Data represent adjusted regression coefficients (β) and negative log_10_-scaled *p* values (−log(*p*)) for the association between metabolites and acute glycaemic dysregulation (presence/absence of glucose in urine), short-term (1,5-AG in plasma) and long-term glycaemic (HbA_1c_) control

^†^Bonferroni significant associations (*p <* 1.8 × 10^−4^ or −log(*p*) > 3.75)

### GGM subnetworks identify key biochemical perturbations associated with diabetes

**Fig. 3:**
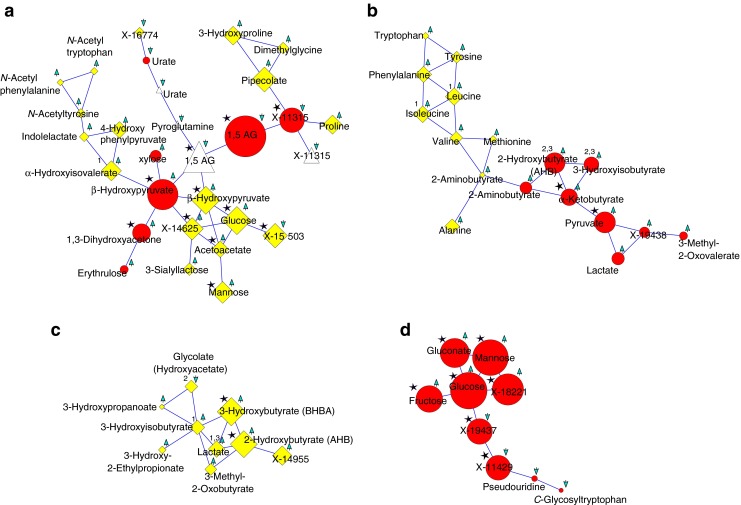
Selected GGM subnetworks. (**a**) 1,5-AG subnet, (**b**) glycolysis–BCAA subnet, (**c**) urinary ketone body subnet, (**d**) carbohydrates subnet. Included are metabolites nominally associated with diabetes (*p* < 0.05); edges indicate significant partial correlations (2.1 × 10^–8^) between two metabolites. Node size is proportional to the absolute β value in the regression analysis with diabetes. Node colour and shape denote the biofluid: white triangle, saliva; red circle, plasma; yellow diamond, urine; arrows indicate the direction of the association (upward, higher in diabetes; downward, lower in diabetes); star indicates an association with all three glycaemic timescales; number indicates an association with glucosuria (1), 1,5-AG (2) or HbA_1c_ (3). For metabolites that are only nominally associated with diabetes, no association with glycaemic control was tested.

We identified 3,742 significant partial correlations (*p <* 2.1 × 10^−8^ after Bonferroni correction) between all 2,178 metabolite measurements, which define the edges between the metabolites in the GGM network (Fig. 1). In total, 1,907 (87.6%) metabolite measures were connected to at least one other metabolite measure by a significant partial correlation edge. For interpretation in the context of this study, GGM nodes were limited to the 546 metabolite measures nominally associated with diabetes (*p <* 0.05) and the edges between these metabolites. This resulted in 33 subnetworks containing at least three nodes, of which 18 subnetworks comprise five or more nodes (see ESM Table 4). Many of the identified subnetworks connect metabolites from the same metabolic pathway (pathway annotation is shown in ESM Table 2). For example, some contain mostly bile acids (subnetworks 7 and 15), medium-chain fatty acids (subnetwork 5), acylcarnitines (subnetwork 12) or carbohydrates (subnetwork 9). Other subnetworks connect metabolites from multiple pathways, such as glycolysis to BCAA metabolism (subnetwork 3). Four of the largest GGM subnetworks are of specific interest for further analysis because they contain many well-established diabetes biomarkers and reflect major pathways known to play roles in diabetes. These four subnetworks (Fig. 3) are: the subnetwork containing 1,5-AG (subnetwork 1, termed 1,5-AG subnet in the following discussion); the subnetwork containing BCAAs and glycolysis-related metabolites (subnetwork 3; glycolysis–BCAA subnet); the subnetwork that includes several urine ketone bodies (subnetwork 8; urinary ketone body subnet); and the subnetwork containing plasma carbohydrates (subnetwork 9; carbohydrates subnet). The complete set of GGM subnets is provided in digital format as ESM Data.

## Discussion

Of the 94 metabolite associations with diabetes found in this study, many have been previously reported in association with diabetes and are confirmed here in a different population. In addition, many metabolites found to be associated with diabetes at a stringent level of significance in one biofluid were also associated at a nominal level of significance in the other biofluid(s). These associations thus provide quality control for the present study and also for the first time a metabolome-wide view of diabetes associations across several body fluids. For instance, perturbations in the glycolysis pathway are reflected by increased pyruvate [23] and lactate levels [24], and perturbations in phenylalanine and tyrosine metabolism have been also found [25]. Increased proteolysis with aminoaciduria is reflected by increased urinary BCAAs and aromatic amino acids [26]. The presence of subclinical ketoacidosis in some patients is indicated by increased levels of 3-hyroxybutyrate and 3-hydroxyisobutyrate [27]. Our study also identified established biomarkers in more than one biofluid, such as 1,5-AG (GlycoMark, GlycoMark, New York, NY, USA) and 2-hydroxybutyrate (Quantose, Metabolon, Durham, NC, USA). The commonly used diabetes drug, metformin, was found to be associated with diabetes in all three biofluids. Of the 16 newly identified metabolite associations, many are in pathways that play a role in diabetes, including β-hydroxypyruvate (glycine, serine and threonine metabolism), 3-methoxytyrosine and 4-hydroxyphenylpyruvate (phenylalanine and tyrosine metabolism), 1,3-dihydroxyacetone (glycolysis pathway) as well as arabitol, gluconate, ribose and xylonate (nucleotide and pentose metabolism), thus linking these metabolites for the first time to diabetes.

Interpretation of large lists of associations can be challenging and requires computational support to place biochemically related metabolites into context. In order to identify biochemical interactions between metabolites and their role in diabetes-related dysregulation, we used Gaussian graphical modelling [15, 16]. Four larger networks are of particular interest and shall be discussed in more detail (Fig. 3). For instance, metabolites in the 1,5-AG subnet reflect the process of limited glucose reabsorption capacity of the kidney in patients with diabetes, linking decreased 1,5-AG levels to elevated urine glucose, pipecolate and proline levels [28], and linking these to other processes, such as ketoacidosis (via the GGM link to acetoacetate and 4-hydroxyphenylpyruvate, a keto acid involved in tyrosine metabolism), perturbed BCAA metabolism (reflected by α-hydroxyisovalerate) and hyperglycemia (reflected in decreased urate levels [29–32]). The glycolysis–BCAA subnet connects metabolites associated with increased proteolysis and aminoaciduria to ketoacidosis (via 3-hyroxybutyrate and 3-hydroxyisobutyrate [27]) and perturbed glycolysis (via pyruvate and lactate). This subnetwork connects the previously reported increased plasma α-ketobutyrate to increased plasma 2-hydroxybutyrate in diabetes [11] by a direct GGM link.

The glycolysis–BCAA and urinary ketone body subnets together highlight the relation of the known diabetes marker 2-hydroxybutyrate [11, 33] with elevated BCAAs, glycolysis and ketoacidosis, which may be interesting for further investigations since 2-hydroxybutyrate is part of the new Quantose clinical test [34]. 3-Hydroxyisobutyrate, known to be associated with ketoacidosis [27] and a product of valine catabolism, is upregulated in both plasma and urine. The links between plasma 3-hydroxyisobutyrate to plasma metabolites of 2-hydroxybutyrate and α-ketobutyrate in the glycolysis–BCAA subnet, in which a set of diabetes predictors are connected (BCAAs, tyrosine, phenylalanine [35] and 2-hydroxybutyrate [33]), and the link between urinary 3-hydroxyisobutyrate to urinary 2-hydroxybutyrate in the urinary ketone body subnet may indicate of a pivotal role for 3-hydroxyisobutyrate in insulin sensitivity and complications associated with diabetes.

Connections between metabolites across the different biofluids were also identified in the GGM subnets. One example is the association of 1,5-AG in plasma and saliva with glucose and ketone bodies (acetoacetate) in urine, as well as to BCAA metabolism in urine (via α-hydroxyisovalerate). Another example is the association of BCAAs, tyrosine and phenylalanine in urine with 2-hydroxybutyrate and ketone bodies in plasma. Moreover, both the glycolysis–BCAA subnet and urinary–ketone body subnet reflected several relationships among metabolites in plasma that were also observed in urine. For example, the association of increased cysteine–methionine metabolism with BCAA metabolism (i.e. GGM link between 2-hydroxybutyrate and 3-hydroxyisobutyrate) in plasma in the glycolysis–BCAA subnet is also seen in urine in the urinary ketone body subnet.

Many of the 16 newly reported markers display a clear biochemical link by GGM edges to known markers of diabetes. β-Hydroxypyruvate is an example of a strong association of a newly reported metabolite with hallmark processes in diabetes. It shows concordant upregulation with diabetes in two biofluids, as in the concordant up- or downregulation of the known markers 1,5-AG, glucose and 2-hydroxybutyrate. In addition, both its plasma and urine metabolites are directly linked in the 1,5-AG subnet to 1,5-AG, and its urine metabolite is directly linked to glucose in urine. This suggests that β-hydroxypyruvate should be further investigated in future studies because it is an intermediate in glucose production from serine [36]. Other molecules such as 4-hydroxyphenylpyruvate and 1,3-dihydroxyacetone also have GGM links to 1,5-AG, ketone bodies and urine glucose in the 1,5-AG subnet. A group of catechols in the tyrosine pathway, namely 3-methoxytyrosine (a product of l-DOPA) [37], is associated with diabetes, possibly reflecting dopamine deficiency, which was previously reported to be associated with visual dysfunction in diabetic rodent models [38]. Also, the links of gluconate to glucose and mannose in the carbohydrates subnet, as well as the link of glycolate to 3-hydroxyisobutyrate in the urinary ketone body subnet, suggest their relevance to diabetes-related metabolic processes represented by these GGM subnetworks. Given the stringent significance cut-off applied in this study, we expect that all 16 associations represent true positives. These may have been seen in this study for the first time because we collected samples from undersampled ethnicities with the potential of displaying very different lifestyles and thus different metabolic patterns.

In order to go beyond mere association with the disease endpoint, we examined how the 94 diabetes-associated metabolites relate to the different timescales of glycaemic control. Our approach of testing these metabolites for a specific association with one or more timescales of glycaemic control can be considered the equivalent of low-, medium- and high-pass frequency filtering. For instance, the levels of a metabolite strongly associated with HbA_1c_ levels but only weakly with glucosuria would be expected to be controlled by biological processes that act on a longer timescale, such as changes in body fat composition. In contrast, metabolites strongly associated with glucosuria but not with HbA_1c_ levels are likely to be involved in biological processes that respond immediately to changes in glucose availability.

Table 4 presents a selection of metabolites that were associated with one or more timescales of glycaemic control. For instance, plasma metabolites that were associated with all three timescales of glycaemic control include pyruvate and 1,3-dihydroxyacetone from the glycolysis pathway; fructose and mannose as carbohydrates and α-ketobutyrate from the cysteine pathway; and β-hydroxypyruvate, gluconate, benzoate and heptanoate (7:0). Urine metabolites that were associated with all three timescales include 3-hydroxybutyrate and acetoacetate as ketone bodies, 1-methylhistidine and *trans*-urocanate from the histidine pathway; xylonate and arabitol as pentose sugars, vanillylmandelate and homovanillate from the phenylalanine and tyrosine pathway; and mannose, 5-oxoproline, kynurenate, *myo*-inositol and β-hydroxypyruvate. Metabolites that are specifically associated with only one or two timescales of glycaemic control include 3-hydroxyisobutyrate and 2-hydroxybutyrate in plasma (associated with short- and long-term glycaemic control but not with acute dysregulation); leucine and isoleucine in urine; and the biochemically related urinary metabolites α-hydroxyisovalerate and 3-hydroxyisobutyrate, of which higher levels are associated with the presence of glucose in urine. Upregulation of malate (Krebs cycle metabolite) in urine was specific to acute dysregulation and short-term (but not long-term) glycaemic control, while higher levels of lactate in urine were specific to long-term (rather than short-term) control. Metabolites that did not show an association with any marker of glycaemic control but were associated with diabetes in the case–control design could be associated with effects of diabetes that are independent of varying glucose homeostasis. Such metabolites include the urine metabolites of phenylalanine, isobutyrylcarnitine, cysteine and alanine, as well as pipecolate in urine and plasma, and metformin in all three biofluids; the latter diabetes drug actually confirms this assumption.

The following limitations of this study need to be considered: (1) patients and controls were not matched for age, sex, ethnicity and BMI. However, adjusting for these factors in the statistical analysis, as we do here, provides an equivalent statistical power to taking a sample-matching approach [39, 40]. (2) All study participants were enrolled at the Dermatology Department of HMC. Most patients were not being treated for acute clinical diabetes dysregulation, so their metabolic state is most likely to represent the average patient with diabetes on a day-to-day basis. Several participants were treated for diseases such as eczema and psoriasis and were taking glucocorticoids or immunosuppressive drugs. Patients with diabetes were taking a wide range and combinations of metabolically active drugs, such as oral hypoglycaemic drugs, insulin and statins. (3) Our participants were in a non-defined fasting state at the time of sample collection. Nevertheless, given the study setting, most participants did not have a major meal at least 2 h prior to sampling and therefore were not acutely postprandial. (4) We collected spontaneous urine samples, rather than acquiring more representative 24 h collections. (5) Diabetes patients have a higher prevalence of different components of the metabolic syndrome that may represent confounding factors. We therefore conducted a sensitivity analysis and demonstrated that the metabolite–diabetes associations reported in Table 3 were robust when lipid traits, waist circumference, WHR or hypertension were adjusted for in the model (ESM Results). (6) Finally, diabetes-associated complications may influence metabolite profiles. However, we showed that the metabolite associations reported in Table 4 are robust when adjusting for heart disease (*n* = 28), kidney disease (*n* = 17), retinopathy (*n* = 68), slow-healing wounds (*n* = 29) and neuropathy (*n* = 26; ESM Results).

By accepting these logistical limitations, patient and control samples could be collected as they became available at the same location, generally in a random pattern and in large numbers, using identical protocols, instruments and study personnel. Some of these limitations probably increased random error in our data, thus biasing our results toward the null, but would not create any spurious signals. Had we tried to collect samples under more ideal conditions of overnight fasting, the number of participants that could be enrolled in this study using the available resources would have been considerably smaller. We therefore feel that our decision to collect samples as they became available represents a valid trade-off regarding the overall achievable statistical power by considerably increasing the number of samples at the cost of increasing random error in the data. The fact that we could detect 94 metabolites associated with diabetes under these conditions underlines the robustness of our findings.

To the best of our knowledge this is the first study of this magnitude to provide a comprehensive association of metabolic pathways with diabetes in three biofluids from the same patients. By going beyond mere associative analyses with disease and looking at more specific disease-related phenotypes (glucosuria, 1,5-AG, HbA_1c_), we could identify particular metabolic networks that were perturbed in diabetes, some of which related to specific timescales of glycaemic control. Notably, this is also one of the first large-scale metabolomics studies of diabetes to include patients from an Arab population. We trust that the markers and associations reported here, as well as the freely available GGM network of diabetes-related metabolic perturbations, will contribute to the growing picture of metabolic changes associated with diabetes, and will improve the functional understanding of the disease with a view of developing new therapeutic approaches and diagnostic tools.

## Electronic supplementary material

ESM Methods (PDF 154 kb)

ESM Results (PDF 381 kb)

ESM Table 1 (XLSX 180 kb)

ESM Table 2 (XLSX 177 kb)

ESM Table 3 (XLSX 169 kb)

ESM Table 4 (XLSX 168 kb)

ESM Data (PDF 33 kb)

## Abbreviations

1,5-AG: 1,5-Anhydroglucitol
BCAA: Branched chain amino acid
GGM: Gaussian graphical model
HMC: Hamad Medical Corporation
WCMC-Q: Weill Cornell Medical College – Qatar
