# Supplementary material for: A systems view of type 2 diabetes-associated metabolic perturbations in saliva, blood and urine at different timescales of glycaemic control
Source: Diabetologia. 2015 Jun 7;58(8):1855–67. doi: 10.1007/s00125-015-3636-2 (PMC4499109; doi:10.1007/s00125-015-3636-2)
Supplement: Supplementary file 1 — (PDF 154 kb) [file 125_2015_3636_MOESM1_ESM.pdf]

## ESM Methods

**Metabolomics.** Metabolic profiling was achieved using ultrahigh-performance liquid-phase chromatography (LC) and gas-chromatography (GC) separation, coupled with tandem mass spectrometry (MS) at Metabolon Inc., using established procedures and technology (Durham, NC, U.S.A.). The technique has been extensively described before [1, 2]. We therefore describe the essential steps of Metabolon's process here.

***Metabolomics data acquisition.*** Samples were shipped on dry-ice to Metabolon, inventoried and immediately stored at -80°C until analysis. Samples were prepared using Metabolon’s standard solvent extraction method and then split into equal parts for GC/MS and LC/MS/MS analysis. Several technical replicate samples were created from a homogeneous pool containing a small amount of all study samples (called “Client Matrix”). Instrument variability was determined as the median relative standard deviation (RSD) for the internal standards that were added to each sample prior to injection into the mass spectrometers. Overall process variability was determined as the median RSD for all endogenous metabolites that were present in all of the "Client Matrix" samples. Metabolites of known structural identity as well as metabolites of unknown structural identity that were detected in the samples are reported.

***Sample handling.*** Samples were assigned a unique identifier and accessioned into the Metabolon Laboratory Information Management System (LIMS). The samples and all derived aliquots were bar-coded and tracked by the LIMS system. Sample preparation was carried out on a MicroLab STAR (Hamilton Robotics). Recovery standards were added prior to the first step in the

extraction process for quality control (QC) purposes. Sample preparation was conducted using a proprietary series of organic and aqueous extractions to remove the protein fraction while allowing maximum recovery of small molecules. Samples were placed briefly on a TurboVap (Zymark Corp., Hopkinton, MA, USA) to remove the organic solvent. Each sample was then frozen, dried under vacuum, and prepared for the appropriate instrument, either LC/MS or GC/MS. For QA/QC purposes, a number of additional samples were included with each day's analysis. Furthermore, a selection of QC metabolites was added to every sample, including those under test. These metabolites were carefully chosen so as not to interfere with the measurement of the endogenous metabolites. Osmolality values in saliva and urine were measured for normalization purposes.

***Liquid Chromatography/Mass Spectrometry.*** The LC/MS portion of the platform used a Waters ACQUITY UPLC and a Thermo-Finnigan LTQ mass spectrometer, equipped with an electrospray ionization (ESI) source and linear ion-trap (LIT) mass analyzer. The dried sample extract was split into two aliquots, and then reconstituted in acidic or basic LC-compatible solvents, each of which contained 11 or more injection standards at fixed concentrations. One aliquot was analyzed using acidic positive ion optimized conditions and the other using basic negative ion optimized conditions in two independent injections using separate dedicated columns (Waters UPLC BEH C18-2.1x100 mm, 1.7 µm). Extracts reconstituted in acidic conditions were gradient eluted using water and methanol both containing 0.1% formic acid, while the basic extracts, which also used water/methanol, contained 6.5 mM ammonium bicarbonate. The MS analysis alternated between MS and data-dependent $MS^2$ scans, using dynamic exclusion, and the scan range was from 80-1000 m/z.

***Gas Chromatography/Mass Spectrometry.*** The samples destined for GC/MS analysis were re-dried under vacuum desiccation for a minimum of 24 hours prior to being derivatized under dried nitrogen using bistrimethyl-silyl-triflouroacetamide (BSTFA). The GC column was 5% diphenyl / 95% dimethyl polysiloxane fused silica column (20 m x 0.18 mm ID; 0.18 um film thickness) with helium as the carrier gas and a temperature ramp from 60° to 340°C over a 17.5-min period. Samples were analyzed on a Thermo-Finnigan Trace DSQ fast-scanning single-quadruple mass spectrometer using electron impact ionization and operated at unit mass resolving power. The scan range was 50-750 m/z. The instrument was tuned and calibrated for mass resolution and mass accuracy on a daily basis.

***Metabolite identification.*** The raw mass spec data files were processed using Metabolon's proprietary analysis software. Metabolites were identified by comparison to library entries of purified standards or recurrent unknown entities. More than 3,500 commercially available purified standard compounds have been acquired and registered into the LIMS for distribution to both the LC and GC platforms for determination of their analytical characteristics. The combination of chromatographic properties and mass spectra gave an indication of a match to the specific compound or an isobaric entity. Additional entities could be identified by virtue of their recurrent nature (both chromatographic and mass spectral). These compounds have the potential to be identified by future acquisition of a matching purified standard or by classical structural analysis. A variety of curation procedures were carried out to ensure that a high quality data set was made available for statistical analysis and data interpretation. The QC and curation processes were designed to ensure accurate and consistent identification of true chemical entities, and to

remove those representing system artifacts, mis-assignments, and background noise. Metabolon data analysts use proprietary visualization software and interpretation software to confirm the consistency of peak identification among the various samples. Library matches for each compound were checked in each sample and corrected if necessary.

## References


[1] Evans AM, DeHaven CD, Barrett T, Mitchell M, Milgram E (2009) Integrated, Nontargeted Ultrahigh Performance Liquid Chromatography/Electrospray Ionization Tandem Mass Spectrometry Platform for the Identification and Relative Quantification of the Small-Molecule Complement of Biological Systems. Analytical Chemistry 81: 6656-6667

[2] Dehaven CD, Evans AM, Dai H, Lawton KA (2010) Organization of GC/MS and LC/MS metabolomics data into chemical libraries. J Cheminform 2: 9
