# Supplementary material for: A systems view of type 2 diabetes-associated metabolic perturbations in saliva, blood and urine at different timescales of glycaemic control
Source: Diabetologia. 2015 Jun 7;58(8):1855–67. doi: 10.1007/s00125-015-3636-2 (PMC4499109; doi:10.1007/s00125-015-3636-2)
Supplement: Supplementary file 2 — (PDF 381 kb) [file 125_2015_3636_MOESM2_ESM.pdf]

ESM Results

Sensitivity analysis with respect to metabolic syndrome related confounding factors. Coexistence of different components of metabolic syndrome can be a confounding factor. In order to evaluate the potential impact of such factors on our diabetes associations we conducted a series of sensitivity analysis as follows: We added the available metabolic syndrome related variables as covariates, one at a time, to our reference model and evaluated the change in the strength of the association between diabetes state and each of the 94 metabolites reported in Table 3 as a function of these variables. To model metabolic syndrome we used the following covariates: waist circumference, waist-to-hip-ratio, hypertension, triglyceride levels, HDL, LDL, and total cholesterol. We note here that the triglyceride and cholesterol levels may be impacted by the undefined fasting state, but we feel that they nevertheless reflect dyslipidemia to the extent needed for a sensitivity analysis. Since some of these variables have missing values, and also since adding additional covariates to a statistical model changes its number of degrees of freedom, a direct comparison of the test statistics would not be informative. Therefore, we analyse changes in the ranking of the metabolite associations with diabetes state. A large drop in the rank after adding a covariate indicates that this association is not driven by the diabetes state itself, but rather by the tested component of metabolic syndrome. The results of this sensitivity analysis are presented below in Supplemental Results Table 1. No major changes in association rank are observed for the associations that we report, the largest drop in rank being observed for the unknown metabolite X-17323 (with hypertension, the drop in rank was by 37 places in a ranked list of 2178 metabolites). This sensitivity analysis confirms the robustness of our associations with respect to metabolic syndrome related covariate (which probably also due to the fact that we only report Bonferroni significant

associations – larger changes in rank are observed for weaker associations, the top association do not change at all).

Supplemental Results Table 1: Rank changes of the 94 associations between a metabolite and diabetes when one of the indicated metabolic syndrome related covariates is added to the linear model. Metabolites are ordered by decreasing strength of association with respect to the reference model (p-values are reported in Table 3 of the manuscript).

METABOLITE	TG[1]	CHOL-ESTEROL[2]	HDL[3]	LDL[4]	WAIST[5]	WAIST-HIP[6]	HYPER-TENSION[6]
1,5-Anhydroglucitol (1,5-AG) (plasma)	0	0	0	0	0	0	0
Mannose (plasma)	0	0	0	0	0	0	0
Glucose (plasma)	0	0	0	0	0	0	0
Metformin (urine)	0	0	0	0	0	0	0
Metformin (plasma)	0	0	0	0	0	0	0
X-18221 (plasma)	0	0	0	0	0	0	0
Gluconate (plasma)	0	0	0	0	0	0	0
Myo-inositol (urine)	0	0	0	0	0	0	0
3-Hydroxybutyrate (BHBA) (urine)	-1	0	0	1	0	0	0
β-Hydroxypyruvate (plasma)	1	0	-1	0	0	0	0
Glucose (urine)	0	0	0	-1	0	0	0
X-17676 (urine)	-1	-3	-1	-2	-1	0	-3
Fructose (plasma)	-1	-1	-1	-2	-1	-1	1
2-Hydroxybutyrate (AHB) (urine)	-1	2	-1	1	2	1	1
1,5-Anhydroglucitol (1,5-AG) (saliva)	3	2	4	3	-3	-1	-2
Pipecolate (urine)	0	-2	0	0	0	-1	-3
X-19437 (plasma)	0	0	0	0	1	-1	2
β-Hydroxypyruvate (urine)	0	1	0	-1	2	3	2
X-15503 (urine)	0	1	0	1	0	0	0
X-11315 (plasma)	0	0	0	0	-1	0	-1
X-11429 (plasma)	0	0	0	-1	1	0	3
X-14331 (urine)	-1	-1	-1	-1	0	-1	-1
Malate (urine)	1	1	1	2	0	1	-1
Mannose (urine)	0	-1	0	-2	0	0	2
5-Oxoproline (urine)	0	1	0	1	0	0	0
Pyruvate (plasma)	0	-1	-1	-1	-1	-2	-1
2-Hydroxybutyrate (AHB) (plasma)	1	5	0	6	-1	-2	-3
Pipecolate (plasma)	1	-2	2	0	-3	-2	-1
X-14625 (urine)	-5	-5	-5	-8	1	1	1
Proline (urine)	1	-3	0	-1	1	3	-5
X-18887 (urine)	2	0	2	2	-4	-4	-1
X-17629 (plasma)	0	-4	-5	-3	-8	-8	6
Vanillylmandelate (VMA) (urine)	-2	1	0	0	1	0	-2
2-Methylcitrate (urine)	-3	4	-2	1	3	2	-4
Homocysteine (urine)	-4	1	-4	-6	3	2	2
Cystine (urine)	4	1	4	2	-4	-4	2

X-18475 (urine)	2	0	0	-2	-3	-3	4
X-11333 (urine)	-1	2	2	0	5	0	6
α-Hydroxyisovalerate (urine)	-7	-4	-7	-5	5	7	-2
X-14955 (urine)	-2	0	0	-1	3	6	2
X-13431 (urine)	1	0	3	-2	6	6	-17
Glycylglycine (urine)	-1	-2	0	-4	4	5	-3
Pro-hydroxy-pro (urine)	-1	2	2	2	-5	-7	-3
X-12682 (urine)	-2	-6	-5	-5	0	2	-5
Adipate (urine)	-1	-2	-3	-4	-1	-1	-10
3-Hydroxyproline (urine)	-2	-6	-6	-2	-13	-19	-5
1,3-Dihydroxyacetone (plasma)	-2	5	4	9	-2	-1	-6
Lactate (urine)	-4	3	-2	-7	7	8	3
Citrulline (plasma)	-6	-7	3	-1	-10	-5	1
α-ketobutyrate (plasma)	4	13	2	16	0	1	8
1-Methylhistidine (urine)	-4	8	-1	3	6	6	8
X-15497 (plasma)	-10	-3	-6	-5	-19	-20	-10
1-Methylhistidine (plasma)	11	3	12	4	5	-1	15
Metformin (saliva)	-6	-9	0	-5	-14	-1	2
Isoleucine (urine)	-7	-3	-7	-10	2	2	17
Acetoacetate (urine)	-4	-2	-5	-22	8	8	7
Leucine (urine)	-15	-2	-18	-9	-14	-10	4
Ribose (urine)	-3	-3	-2	-5	1	4	-10
X-10593 (urine)	0	-10	-6	-6	-5	-2	-3
X-11540 (plasma)	-6	0	1	5	-3	-5	-4
Heptanoate (7:0) (plasma)	21	-25	-2	-14	-12	-11	8
4-Hydroxyphenylpyruvate (urine)	-3	-26	-8	-14	1	2	-8
Phenylalanine (urine)	2	2	-1	-2	-7	-4	6
Threonate (urine)	-10	5	-15	1	7	8	-8
Creatinine (urine)	-7	8	-4	4	-4	-2	3
Ethanolamine (urine)	-7	11	-5	-8	-12	-30	14
γ-Glutamylleucine (urine)	-12	-19	-17	-20	-6	-16	10
X-17299 (urine)	-4	8	1	8	7	5	4
Pyroglutamine* (plasma)	-4	5	-1	14	8	11	3
Arabitol (urine)	-4	5	-2	3	1	-1	-1
3-Hydroxyisobutyrate (plasma)	-14	0	-14	4	-19	-16	-12
7-Ketodeoxycholate (urine)	-4	4	-1	2	19	27	7
Homovanillate (HVA) (urine)	-7	5	1	5	-17	-9	-21
X-12253 (urine)	-16	-1	-8	7	-2	-5	-17
Isobutyrylcarnitine (urine)	14	8	12	13	19	8	-27
Trans-urocanate (urine)	-14	4	-15	5	-17	-17	1
X-12170 (urine)	2	-11	11	-4	-1	8	-16
Alanine (urine)	-1	2	3	3	3	-5	3
Pro-hydroxy-pro (plasma)	2	-2	10	-4	-5	-10	-15

Ornithine (urine)	1	-8	8	-5	-1	4	5
Homocitrulline (urine)	10	7	11	10	10	11	-11
X-11315 (saliva)	-5	-14	0	-1	-15	-4	-11
Kynurenate (urine)	-11	15	2	11	17	8	-2
X-17323 (urine)	-6	-11	-12	-10	15	13	-37
Benzoate (plasma)	28	-18	-8	-8	-14	-3	30
Xylonate (urine)	-10	2	-14	-6	0	-2	-21
X-13840 (urine)	5	-18	9	-15	3	10	-4
3-Hydroxyisobutyrate (urine)	-13	-4	-10	1	-4	4	20
N1-Methyladenosine (urine)	-12	12	-3	12	1	-3	4
N-Acetyl-β-alanine (urine)	-10	-21	-5	-25	9	0	0
3-Methoxytyrosine (plasma)	-25	9	-8	1	-10	-8	20
γ-Glutamylglutamine (plasma)	5	-3	5	9	-27	-22	0
Glutamate (urine)	-12	-8	-9	-18	1	0	1
Glycolate (hydroxyacetate) (urine)	14	16	17	6	6	-5	-28

[1]Triglyceride levels, [2]total cholesterol, [3]HDL cholesterol, [4]LDL cholesterol, [5]waist circumference, [6]waist-hip ratio, [7]hypertension

Sensitivity analysis with respect to diabetes-associated complications. Metabolite profiles may be influenced by the presence of diabetes-associated complications. We conducted a sensitivity analysis as described above on the available variables related to diabetes-associated complications, adding one covariate at a time and reporting the relative change in the rank of the association. As shown in Supplemental Results Table 2 below, most associations were robust with respect to the influence of self-reported heart disease (N=28), kidney disease (N=17), retinopathy (N=68), slow healing wounds (N=29), and neuropathy (N=26).

Supplemental Results Table 2: Sensitivity analysis on the effect of diabetes-associated complications. The table reports rank changes of the association between a metabolite and the indicated glycemic control timescale when one of the indicated covariates is added to the linear model. (Limited to metabolites from Table 4 of the manuscript, from a ranked list of 2178 metabolites).

Time-scale[1]	Metabolite	-Log (p)[2]	Heart disease	Kidney disease	Retino-pathy	Slow healing wounds	Neuro-pathy
acute	3-Hydroxyisobutyrate (urine)	5.3	-19	-7	-15	-6	-15
acute	Isoleucine (urine)	5.4	-12	-2	-7	-11	-12
acute	Leucine (urine)	5.9	-34	-31	-34	-24	-30
acute	α-Hydroxyisovalerate (urine)	6.6	-6	7	-8	-9	-9
acute	Pyruvate (plasma)	8.5	-3	-2	-6	2	-10
acute	Lactate (urine)	9.7	-4	4	-1	-3	-2
acute	3-Hydroxybutyrate (BHBA) (urine)	12.5	-15	-6	-12	-13	-13
acute	2-Hydroxybutyrate (AHB) (urine)	15.4	-2	4	-1	0	-2
acute	Acetoacetate (urine)	18.8	0	-1	0	0	0
acute	Mannose (urine)	20.1	2	-1	2	3	2
acute	β-Hydroxypyruvate (urine)	35.1	0	0	0	0	0
acute	Glucose (urine)	X	X	X	X	X	X
short	Glycolate (hydroxyacetate) (urine)	4.9	25	21	16	16	21
short	3-Hydroxyisobutyrate (plasma)	5.7	-20	-24	-19	-28	-28
short	α-Ketobutyrate (plasma)	5.5	-10	-32	-29	-30	-23
short	2-Hydroxybutyrate (AHB) (plasma)	10.2	4	-2	0	-1	-2
short	1,5-Anhydroglucitol (1,5-AG) (saliva)	18.6	0	1	1	0	0
short	1,5-Anhydroglucitol (1,5-AG) (plasma)	X	X	X	X	X	X
long	1,3-Dihydroxyacetone (plasma)	6.5	-16	-16	-4	2	-14
long	Fructose (plasma)	15.9	0	0	-1	0	-1
long	β-Hydroxypyruvate (plasma)	18.0	0	0	1	0	1
long	Gluconate (plasma)	18.9	0	0	-1	0	-1
long	Glucose (plasma)	26.4	0	0	0	1	0
long	Mannose (plasma)	27.5	0	0	0	-1	0

[1]glycemic timescale, [2] -log10(p-value) for the association with the corresponding glycaemic timescale
