# Supplementary figures and images for: A systems view of type 2 diabetes-associated metabolic perturbations in saliva, blood and urine at different timescales of glycaemic control
Source: Diabetologia. 2015 Jun 7;58(8):1855–67. doi: 10.1007/s00125-015-3636-2 (PMC4499109; doi:10.1007/s00125-015-3636-2)

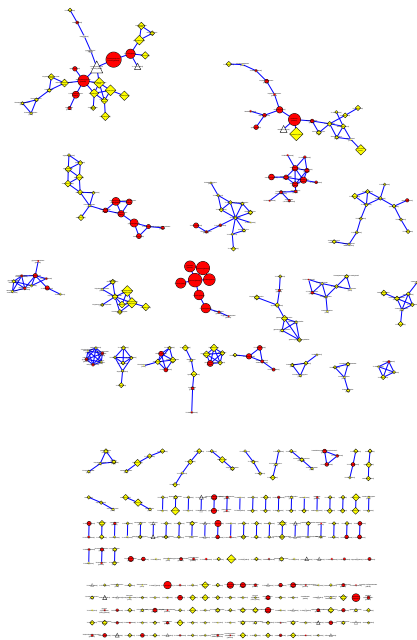

Supplement: Supplementary file 7 — (PDF 33 kb) [file 125_2015_3636_MOESM7_ESM.pdf]
